# Observational evidence of stratification control of upwelling and pelagic fishery in the eastern Arabian Sea

**DOI:** 10.1038/s41598-021-86594-4

**Published:** 2021-03-31

**Authors:** Jayu Narvekar, Riyanka Roy Chowdhury, Diksha Gaonkar, P. K. Dinesh Kumar, S. Prasanna Kumar

**Affiliations:** 1 CSIR-National Institute of Oceanography, Dona Paula, Goa 403004 India; 2 Centre for Oceans, Rivers, Atmosphere and Land Sciences, Indian Institute of Technology Kharagpur, Kharagpur, West Bengal 721302 India; 3 CSIR-National Institute of Oceanography Regional Centre, Dr. Salim Ali Road, Kochi, 682018 India

**Keywords:** Marine biology, Physical oceanography, Ocean sciences

## Abstract

Upwelling is a physical phenomenon that occurs globally along the eastern boundary of the ocean and supports pelagic fishery which is an important source of protein for the coastal population. Though upwelling and associated small pelagic fishery along the eastern Arabian Sea (EAS) is known to exist at least for the past six decades, our understanding of the factors controlling them are still elusive. Based on observation and data analysis we hypothesize that upwelling in the EAS during 2017 was modulated by freshwater-induced stratification. To validate this hypothesis, we examined 17 years of data from 2001 and show that inter-annual variability of freshwater influx indeed controls the upwelling in the EAS through stratification, a mechanism hitherto unexplored. The upper ocean stratification in turn is regulated by the fresh water influx through a combination of precipitation and river runoff. We further show that the oil sardine which is one of the dominant fish of the small pelagic fishery of the EAS varied inversely with stratification. Our study for the first time underscored the role of freshwater influx in regulating the coastal upwelling and upper ocean stratification controlling the regional pelagic fishery of the EAS.

## Introduction

**Figure 1:**
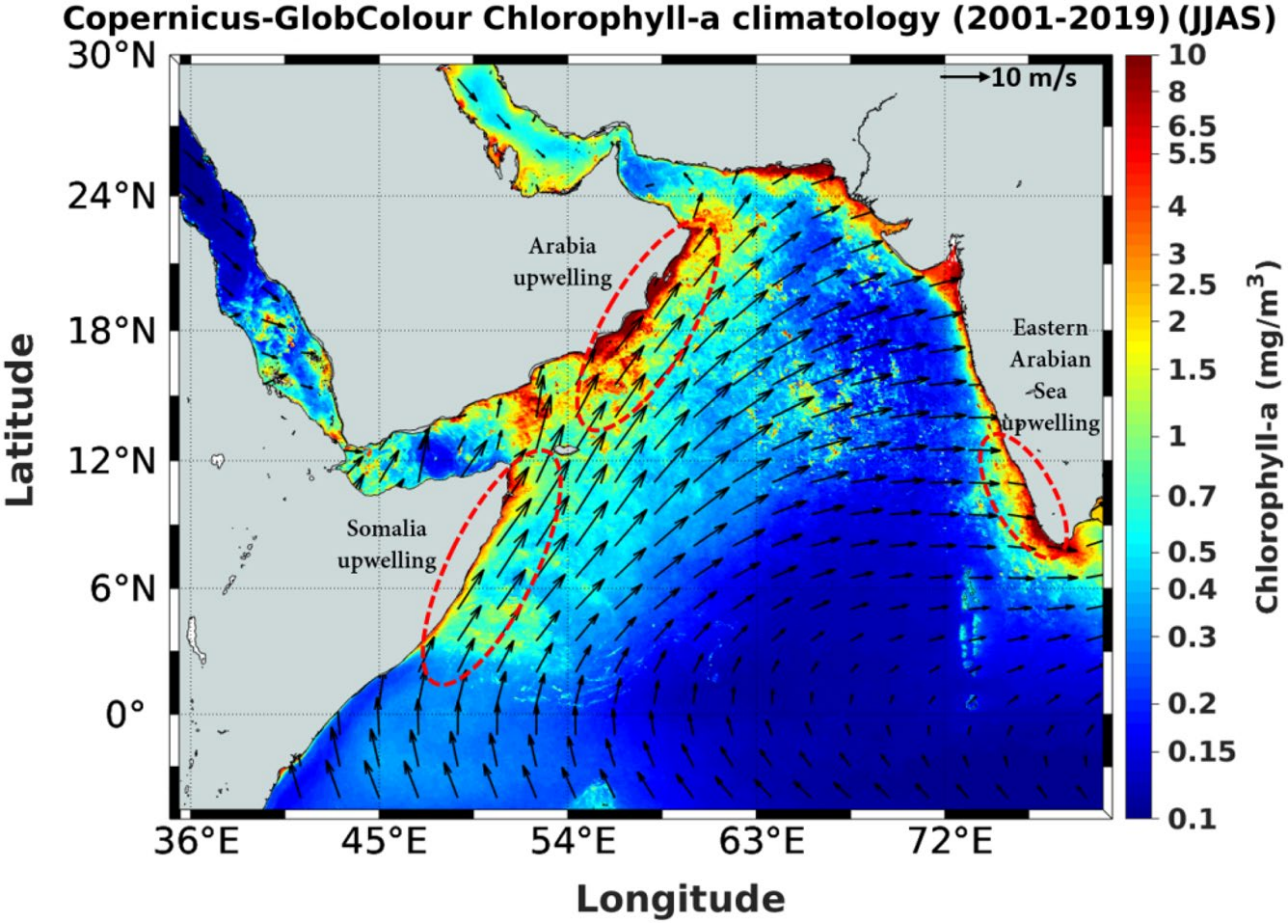
Map of the Arabian Sea showing the coastal upwelling regions off Somalia, Arabia and southern part of the EAS demarcated by red broken ellipse. Shading is the seasonal climatology (June–September) of chlorophyll *a* (Chl-a) pigment concentration (mg/m^3^) derived from Copernicus-GlobColour merged product overlaid with climatological (June–September) wind vectors obtained from MERRA, both for 2001 to 2019. Figure was generated using MATALAB software. See text for details.

Upwelling is a physical process that brings cold and nutrient-rich subsurface waters to the upper ocean. This process makes the nutrient-deficit and sunlight-replete waters of the upper ocean biologically productive by kick-starting the organic carbon production by phytoplankton through photosynthesis. Consequently, upwelling regions of the world ocean are also regions of intense fishery activities^1^. Though upwelling regions occupy only 5% of the total ocean area, it accounts for 25% of the global marine fish catch^2^. Usually, eastern boundaries of the ocean are well known for coastal upwelling^3^ and high chlorophyll *a* (Chl-a) concentrations, such as California, Peru and Chile coasts in the Pacific and West Africa (Canary upwelling) and South-West Africa (Benguela upwelling) in the Atlantic. The exception is the coastal upwelling along Somalia^4^ and Arabia^5^ in the Indian Ocean that occurs along the western boundary (Fig. 1). This is because the seasonal monsoon wind that blows in a south-westerly direction (summer or south-west monsoon) from June to September drives offshore Ekman transport supporting upwelling. The same south-west monsoon wind system also drives upwelling along the eastern Arabian Sea (EAS)^6,7^ (Fig. 1). This makes the Arabian Sea a unique basin with seasonal upwelling occurring along both eastern and western boundaries. Though the magnitude of upwelling in the EAS is much smaller compared to the other eastern boundary upwelling regions, it supports a substantial pelagic fishery like oil sardine, mackerel, and anchovies^8–13^ that supplements the nutrition requirement of the population in this region. In fact, the pelagic fishery from the EAS contributes to 20% of the total marine fish catch from India^14^, while the oil sardine alone accounts for 15% of the total marine fish landings of India^15^.

**Figure 2:**
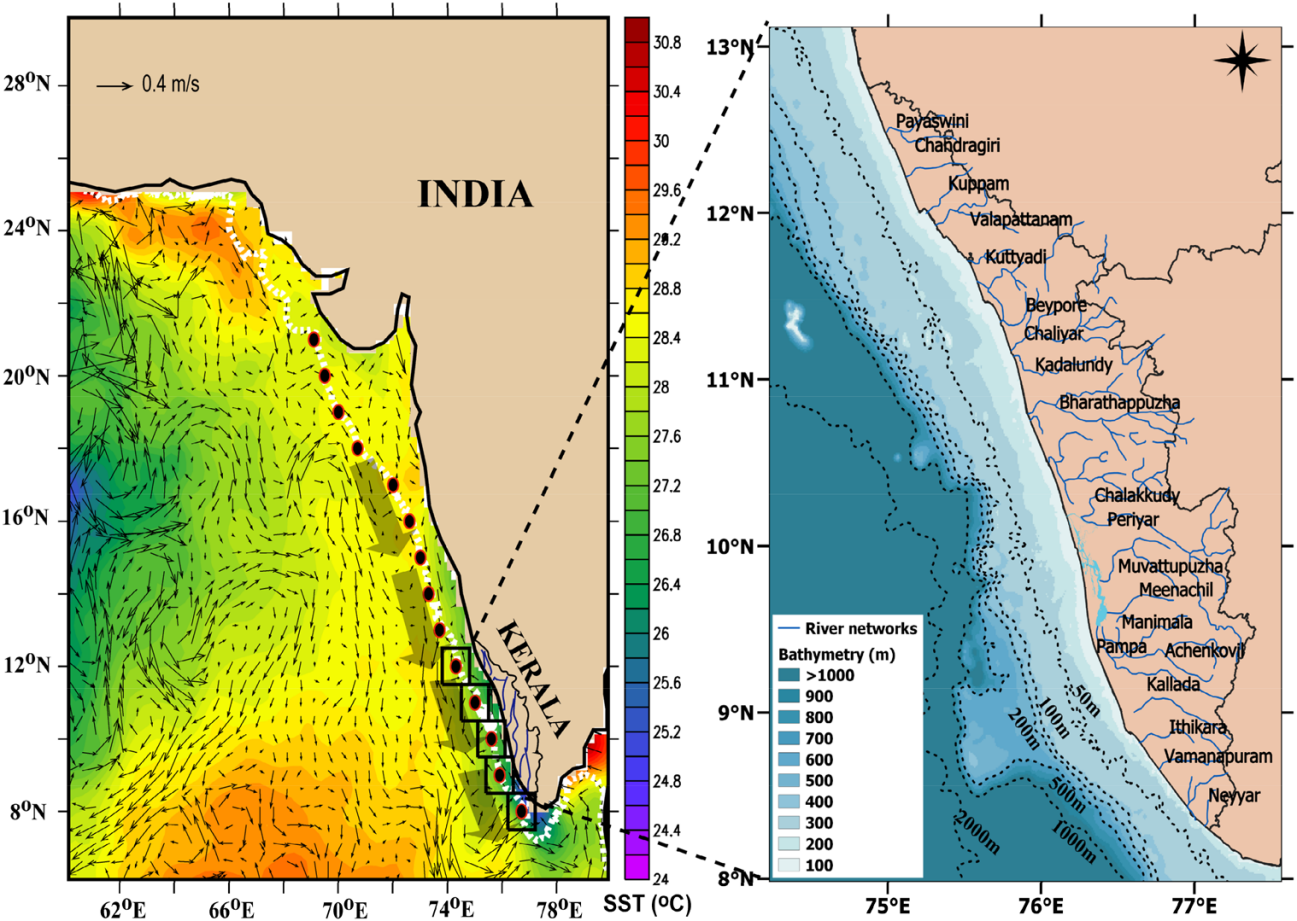
Location map of the eastern Arabian Sea showing the study area (left panel). Red circles filled with black are the station locations from where in situ water column data were collected during 3rd to 28th August 2017. The white dashed line represents the 200 m depth contour. Shading is the sea surface temperature (SST, °C) and overlaid vectors represent the geostrophic current both for August 2017 obtained from NOAA and CMEMS respectively. The black squares are the boxes centered at in situ stations from 8° to 12° N used for the calculation of time series of various parameters. The wide grey-shaded arrows along the EAS represent the West India Coastal Current (WICC). The river network map of Kerala along with bathymetry is also given (right Panel). Figure was created using using Ferret and QGIS software. See text for details.

The regional oceanography of the EAS is unique^16^ with semi-annually reversing winds and coastal currents^17^, upwelling driven phytoplankton bloom and primary production^6^, seasonal hypoxia^18^, intrusion of high and low salinity waters^19,20^, presence of coastal Kelvin wave and westward propagating Rossby wave^17^, and occurrence of warm pool^21^. The coastal regions of the EAS receive substantial seasonal monsoon rainfall from June to September, which feeds the numerous small and medium rivers flowing through the coastal plain and joins the Arabian Sea (Fig. 2, right panel). The prevailing coastal current, the West India Coastal Current (WICC), at this time of the year flows southward.

The upwelling along the EAS is a seasonal phenomenon, the report of which dates back to 1959^6^. There had been several studies aiming at understanding the process of upwelling based on water column data^7,22–24^. The above studies suggest that upwelling in the EAS starts towards the end of May with the reversal of wind from north-easterly direction to south-westerly direction, intensifies and persists during June to August, starts diminishing by September, and ceases completely by October when the south-westerly wind collapses. Spatially, upwelling is most intense and active in the southern part of EAS along the littoral state of Kerala (Fig. 2), appearing first in the southernmost latitude off Kanyakumari (8° N) towards the end of May, slowly progresses northward with the advancement of south-west monsoon winds and extends up to the coast of Ratnagiri (17° N) by August^22,25,26,30^. Previous studies suggest that upwelling in the EAS is driven by a combination of local forcing through wind^7,27,28^ and remote forcing via coastal Kelvin wave^17,22,29–32^. In spite of the above mentioned studies our understanding of upwelling along the EAS is incomplete. Even lesser is our knowledge on its inter-annual variability with only two studies^33,34^ both of which speculated wind as the most probable cause. Though there had been some attempts to unravel the relationship between upwelling and small pelagic fishery of the EAS^13,14,26,35^, the role of regional ocean processes on fishery still remains elusive.

The above motivated the present study and prompted us to ask the following question: What is the role of atmospheric drivers and ensuing ocean processes in regulating the upwelling and regional small pelagic fishery of the EAS? We seek answer to the above question by examining (1) whether there are any forcing other than wind and coastal Kelvin wave that could bring about changes in the process of upwelling, and (2) if so, can such forcing explain the observed inter-annual variability in upwelling and its connection to small pelagic fishery of the EAS. Using a combination of in situ as well as remote sensing data we show that the upper ocean stratification induced by freshwater input is an important forcing that modulates upwelling in the EAS in 2017. It is further shown that the upwelling at inter-annual time scale is regulated by the variability in rainfall, river discharge and oceanic precipitation, a result which is so far unexplored. Finally, we show a mechanistic relationship between oil sardine, a small pelagic fish that dominates the EAS upwelling region, and water column stratification at inter-annual time scale.

## Results and discussion

The water column temperature, salinity, and static stability from 8 to 21° N along the EAS were examined to understand the vertical structure and latitudinal extent of upwelling during August 2017. Thereafter, the latitudinal variability of the depth of 24 °C isotherm (D_24_, a proxy for upwelling), upper water column (0–1 m) static stability (an indicator of stratification) and wind forcing via Ekman vertical velocity and Ekman mass transport (EMT) were analysed to infer about the forcing that controls upwelling process. Subsequently, the rainfall and cumulative discharge from 21 major rivers and oceanic precipitation in the southern part of the EAS during 2001 to 2019 were analysed along with inter-annual variability of D_24_ and oil sardine data to understand the role of stratification in controlling the oil sardine fishery.

### Vertical structure of water column parameters

**Figure 3:**
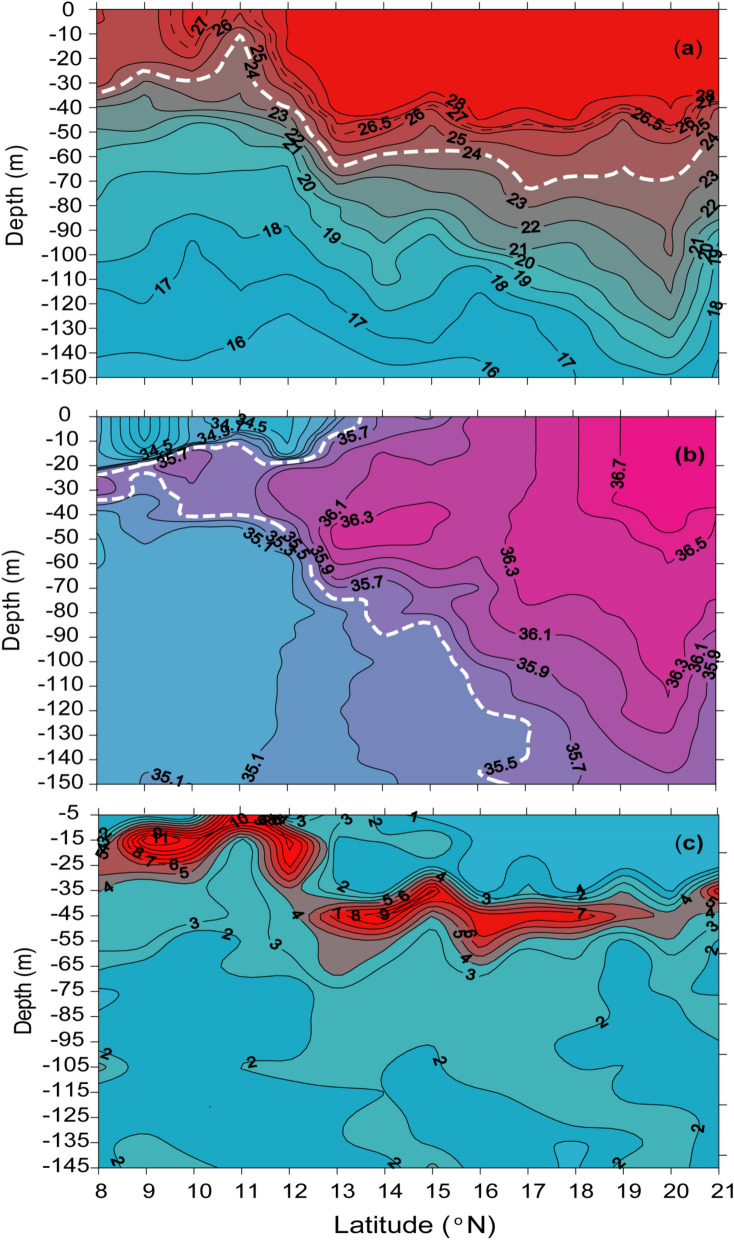
Vertical distribution of (**a**) temperature (°C), (**b**) salinity and (**c**) static stability (E, m^−1^) from 8° to 21° N along the eastern Arabian Sea during August 2017. The white broken line in (**a**) is the 24 °C isotherm, while that in (**b**) is the 35 isohaline.

The salient feature of the vertical thermal structure was the gradual tendency of up-sloping of isotherms from 8 to 10° N, and a sharp up-sloping of isotherms up to 11° N, followed by an equally sharp down-sloping of isotherms up to 13° N (Fig. 3a). For example, the 24 °C isotherm (white broken line in Fig. 3a) which was at 35 m at 8° N shoaled to 10 m at 11° N and then deepened to 65 m at 13° N. A thick isothermal layer of almost uniform thickness of 40 m was noticed north of 13° N, where the temperature ranged from 28.5° to 28 °C. However, the 26.5 °C (black broken line in Fig. 3a) and 27 °C isotherms broke into the surface in the region between 10° and 11° N, indicating the signature of active upwelling. In general, the temperature of the upper 10 m water column south of 11° N was colder than 27 °C, while that in the north was warmer than 28 °C. Thus, from the vertical temperature distribution, we infer that the upwelling was active only in the vicinity of 11° N, where the surfacing of isotherms with colder waters was noticed. In fact, the depth of the 24 °C isotherm could serve as an indicator of upwelling. Based on earlier studies^22,26^ we expected the upwelling to be most active in the EAS from 8° to 13° N. However, in the present study active upwelling was noticed only at 11° N, while in the region north and south of it indicated suppression of upwelling. Based on the latitudinal extent of the shallow D_24_, we infer that the tendency of upwelling extended up to 13° N. In order to further understand why active upwelling was confined in the vicinity of 11° N in August 2017, the vertical salinity distribution was examined.

The vertical salinity structure showed the presence of low salinity waters in the upper water column between 8° and 13.5° N (Fig. 3b) with the lowest salinity of 34.1 occurring at 9° N. North of 13° N the salinity showed a gradual increase with latitude. Note the thick layer of high salinity (36.7) in the upper 50 m between 19° and 21° N and the subsurface salinity maxima (36.3) between 13° and 15° N, which are the signature of the Arabian Sea high salinity water mass^19^. In the south, the thickness of the layer of low salinity water decreased from 8° to 13° N. For example, the 35.5 isohaline (the upper white broken line in Fig. 3b), which is taken as the boundary of low salinity water, was 20 m deep at 8° N. It shoaled to 10 m at 11° N and deepened once again, finally surfaced between 13° and 14° N indicating the northernmost limit of the low salinity water. Similar low salinity values of 34.2 and 34.8 were reported off Cochin (9.9° N) and Kanyakumari (8° N) respectively in August based on measurements^36^. In the present case, the observed pattern of distribution of low salinity water (Fig. 3b) was consistent with temperature (Fig. 3a). Recall the surfacing of isotherms between 10° and 11° N indicating the upwelling, which also should bring subsurface high salinity waters to the surface. An indication of this is discernible in the salinity distribution where surfacing of 34.7 isohalines were seen between 10° and 11° N. The presence of low salinity water in the upper water column could potentially impart strong stratification. Hence, to ascertain this aspect we analysed the static stability of the water column which is a measure of stratification.

The vertical distribution of static stability (E, m^−1^) showed high values with strong gradient confined within upper 30 m between 8° and 13° N, and below this depth the values decreased rapidly (Fig. 3c). North of 13° N, a region of strong gradient in static stability was located between 30 and 60 m, while below this depth values decreased. The first region with high static stability located in the upper 30 m of the water column in the south between 8° and 13° N coincided with the region of low salinity waters. This clearly indicated the role of freshwater in controlling the stability of the upper water column and is the signature of haline stratification. The second region with high static stability located in the sub-surface layer of the water column north of 13° N coincided with the upper thermocline. This is the manifestation of thermal stratification. Note that at 11° N the static stability was the least with nearly the same value in the upper 10 m and the isolines of the static stability outcropped into the surface. It is appropriate to mention here that though it is the freshwater influx that dominantly controls the stratification in the upper water column in the southern part of the EAS, the temperature of the upper thermocline also contributed to the overall stratification.

### Latitudinal variability of wind-driven Ekman dynamics

**Figure 4:**
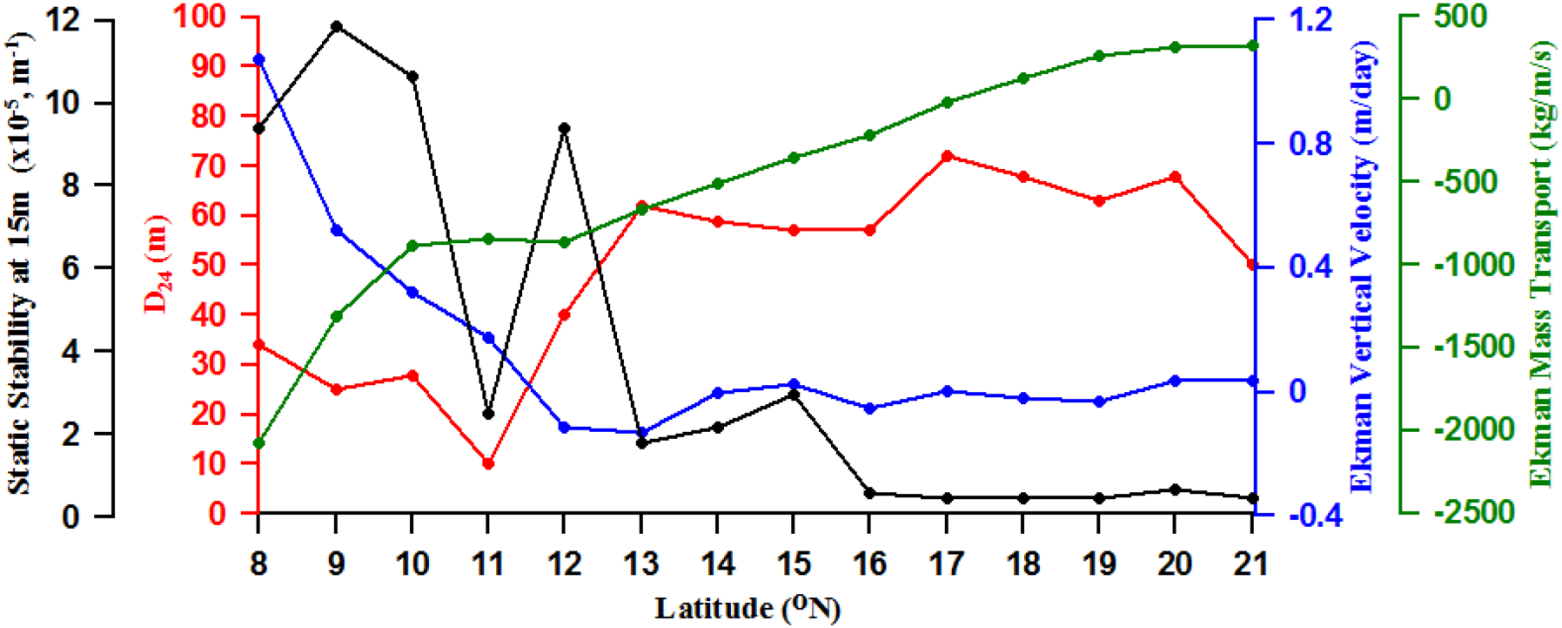
Latitudinal variation of Ekman mass transport (EMT) (kg/m/s, green solid line), Ekman vertical velocity (m/day, blue solid line), static stability in the upper 15 m of the water column (m^−1^, black solid line), and depth of 24 °C isotherm (D_24_) (m, red solid line) from 8° to 21° N along the eastern Arabian Sea during August 2017.

In spite of the presence of fresh water-driven stratification between 8° and 13° N, the upwelling was active at 11° N. Since the coastal wind is one of the factors that is known to modulate the upwelling in the EAS^7,22^, the latitudinal variation of wind-driven Ekman mass transport (EMT), Ekman vertical velocity, static stability in the upper 15 m of the water column, and D_24_ at stations from 8° to 21° N (Fig. 4) were examined. We used D_24_ instead of the commonly used D_26_ as an indicator of upwelling, though both lie in the upper thermocline, due to the following reason. During upwelling the 26 degree isotherm in the southern part of EAS often outcrops and hence has the potential of being influenced by the local air-sea fluxes. EMT showed the highest negative value of 2000 kg/m/s at 8° N and represents an offshore transport (negative) of water favouring upwelling. The EMT rapidly reduced to less than half its value at 10° N and remained almost uniform up to12° N. Beyond this latitude the EMT decreased rapidly becoming zero at 17° N, which indicated the northward limit of potential upwelling. Between 18 and 21° N the EMT was small but onshore (positive) indicating condition favourable for sinking. Commensurate with EMT, the Ekman vertical velocity was highest (1.1 m/day) and positive (Ekman suction) at 8° N. Beyond this latitude, the Ekman vertical velocity though decreased rapidly, remained positive up to 11° N (0.2 m/day), which indicated the conditions favourable for upwelling. However, from 12° to 21° N the values were close to zero or small and negative (Ekman pumping) indicating neutral condition or condition suitable for sinking. In spite of substantial positive Ekman vertical velocity and large offshore EMT from 8 to 11° N, the thermal structure did not show the signature of active upwelling in this region, except at 11° N (Fig. 2a). The D_24_, though showed a weak shoaling tendency from 8° to 10° N was deeper than 25 m. The static stability in the upper 15 m of the water column at these latitudes was high with values ranging from 10 to 12 × 10^–5^ m^−1^ indicating strong stratification. However, at 11° N the D_24_ was the shallowest (10 m) and the static stability showed a rapid decrease to 2 × 10^–5^ m^−1^. This point to the fact that the strong upwelling observed at 11° N was driven by the reduction of the stratification of the water column. Recall that the thickness of low salinity water was the least at 11° N (Fig. 3b) and this led to the decrease in static stability and stratification of the upper water column. North of 11° N the D_24_ deepened sharply to 60 m at 13° N and remained uniform until 21° N. Both D_24_ and static stability showed that the northern limit of the upwelling tendency was 13° N. Hence, our inference is that the prevailing winds were able to create a condition favourable for upwelling from 8^o^ to 12° N through potential offshore EMT and subsequent Ekman suction. However, the presence of low salinity water in the upper 20 m of the water column increased the static stability and stratification. This inhibited the process of upwelling except at 11° N where the thickness of the low salinity layer was the least. Though wind-forcing was favourable for upwelling in the region from 8° to 11° N, the freshwater flux driven upper ocean stratification counteracted it. However, at 11° N the wind-forcing was able to overwhelm the stratification. It is important to note that upwelling has considerable spatial variability within the EAS due to the variability in wind as well as the freshwater influx, both of which are strongly related to the south-west monsoon.

It is amply clear from the above that the presence of low salinity water in the upper water column in the southern part of the EAS played an important role in modulating upwelling in August 2017 through stratification (static stability). The source of freshwater could either be river runoff or oceanic precipitation or both. In the next section, we examine the role of both river runoff and oceanic precipitation in controlling the upwelling.

### Rainfall, river discharge and upwelling

**Figure 5:**
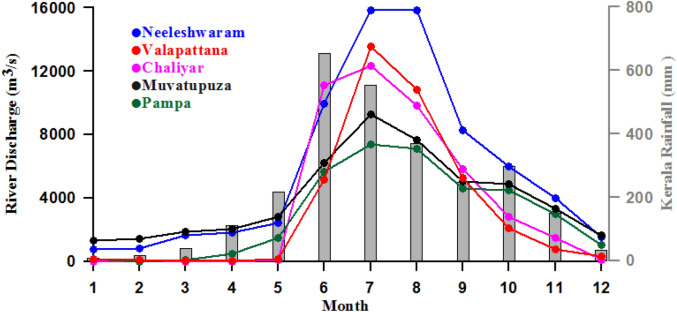
Monthly mean climatology of rainfall (2001–2019) (mm, grey bar) and river discharge (2001–2018) (m^3^/s) from 5 major rivers (solid lines) of the littoral state of Kerala, India.

The littoral state of Kerala (8°18′ N and 12°48′ N latitude and 74°52′ E and 77°2′ E longitude; See Fig. 2, right panel) adjoining the southern part of the EAS has 41 west-flowing small rivers that join the Arabian Sea. These rivers are rain-fed with an annual average rainfall of 3000 mm^37^. To understand the seasonal cycle of river discharge in the context of the seasonal rainfall, the monthly mean climatology (2001–2018) of five major rivers and rainfall of Kerala (2001–2019) are presented in Fig. 5. The Kerala rainfall showed an annual cycle with rainfall increasing from January, attaining the highest value in June (650 mm) and then declining rapidly to December. The months from June to October, representing the south-west monsoon period, accounts for the bulk of the annual rainfall. The discharge from the five major rivers also showed a similar annual cycle commensurate with that of rainfall with most of the discharges during the south-west monsoon months. Note that the peak discharge was in July, a month after the peak in rainfall. Thus, it is evident that the rainfall driven river discharge which peaks during south-west monsoon could contribute to the freshening of surface water off the littoral state of Kerala between 8° and 12° N thereby increasing the stratification of the water column and impacting the upwelling. Further confirmation of the role of rain fall, river discharge, and stratification using multiple linear regression analysis is presented in Table 1 and discussed in subsequent paragraph.

**Table 1:** Multiple linear regression analysis with de-trended D_24_ as the predictand and parameters such as oceanic precipitation, river discharge, and Kerala rainfall as predictors for the period 2001–2019.

Predictor	Rank	Regression coefficient	Standard error	*t*	*p* >|*t|*
Oceanic precipitation	1	0.682	0.183	3.733	0.002*
River discharge	2	0.663	0.187	3.547	0.003*
Kerala rainfall	3	0.626	0.195	3.214	0.005*

*Denotes the values that are statistically significant at *p* < 0.05.

**Figure 6:**
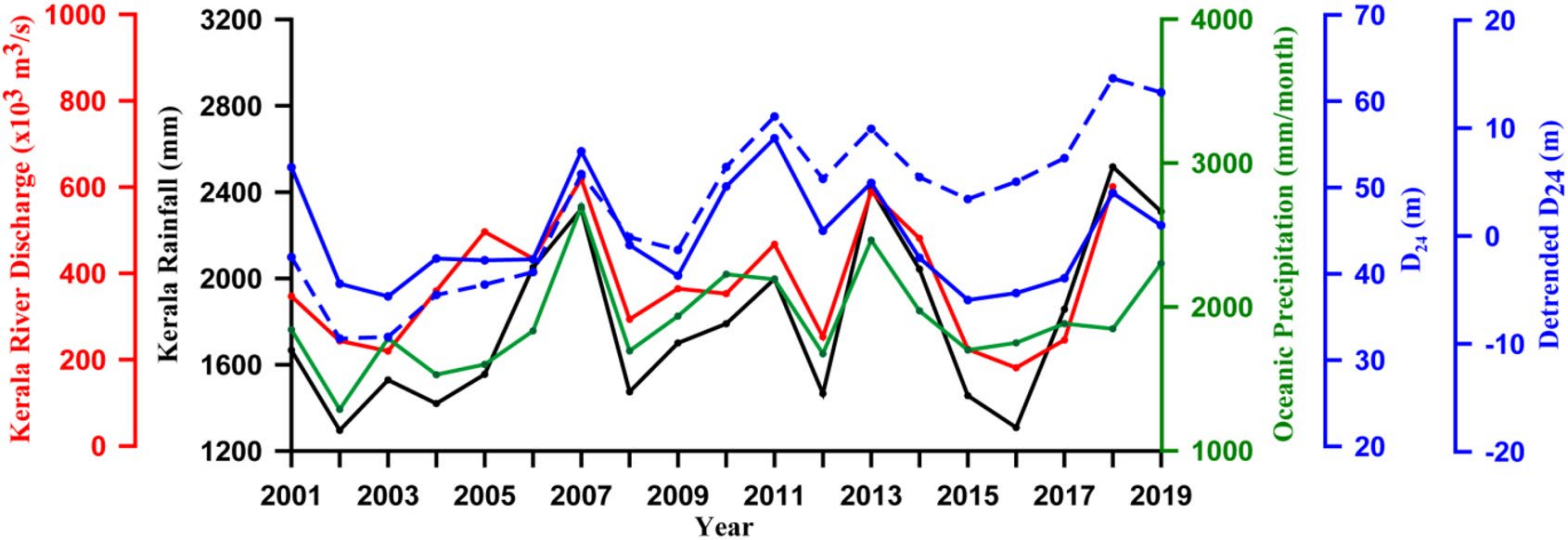
Time series of rainfall (mm, black solid line) and river discharge (× 10^3^ m^3^/s, red solid line) from Kerala, oceanic precipitation (mm/month, green solid line), depth of 24 °C isotherm (D_24_) (m, blue broken line), and de-trended depth of 24 °C isotherm (m, blue solid line) from 2001 to 2019, except for river discharge which is up to 2018. The river discharge and rainfall from Kerala were cumulated for June to September, while the oceanic precipitation and D_24_ were averaged for June to September and also over the boxes from 8° to 12° N. See Fig. 2 for location of boxes.

To ascertain the role of freshwater input in controlling the upwelling through increased stratification on inter-annual time scale, the discharge of 21 major rivers and rainfall in the littoral state of Kerala along with oceanic precipitation and D_24_ were examined. The river discharge and Kerala rainfall were cumulated for each year from June to September, while the oceanic precipitation and D_24_ were averaged from June to September and also over the boxes from 8° to 12° N, and presented for the period from 2001 to 2019 (Fig. 6). Since the time variation of D_24_ showed a trend, the de-trended D_24_ was also presented in Fig. 6. Note that the boxes from 8° to 12° N lie in the core upwelling region in the southern part of the EAS.

The salient feature of the time series (Fig. 6) was the co-variation of Kerala rainfall, river discharge and the oceanic precipitation (referred as freshwater influx) with D_24._ The D_24_ / de-trended D_24_ showed a strong relationship with freshwater influx. For example, during 2001, 2007, 2011, 2013, and 2018 when freshwater influx showed high values, both D_24_ and de-trended D_24_ also showed high values (Fig. 6). The deepening of D_24_ / de-trended D_24_ implied weakening of upwelling. In a similar way, when freshwater influx was low during 2002, 2008, 2012 and 2016, the D_24_ also was low. Shoaling of D_24_ implied strengthening of upwelling. Both cases cited above implied a strong inverse association of upwelling with freshwater influx on inter-annual time scale. A similar inverse relation of upwelling and rainfall was reported from Benguela upwelling region^38^. In another study the changes in the coastal sea level in the Gulf of Alaska was found to be related more strongly to precipitation and runoff rather than changes in wind stress^39^.

To further elucidate the relationship between upwelling and freshwater influx, a multiple linear regression analysis^26^ was performed to assess the relationship between three predictors and a predictand. The de-trended D_24_ was taken as the predictand and three parameters such as oceanic precipitation, river discharge, and Kerala rainfall were taken as predictors for the period 2001 to 2019. The predictors were ranked on the basis of the magnitude of their regression coefficient presented in Table 1. Oceanic precipitation was the most important predictor (ranked 1), followed by river discharge, and then Kerala rainfall (ranked 3). All the three predictors were significant at 95%. Note that there was not much difference among the 3 predictors indicating that on inter-annual time scale oceanic precipitation, river discharge and Kerala rainfall were almost equally and positively correlated with D_24_.

**Figure 7:**
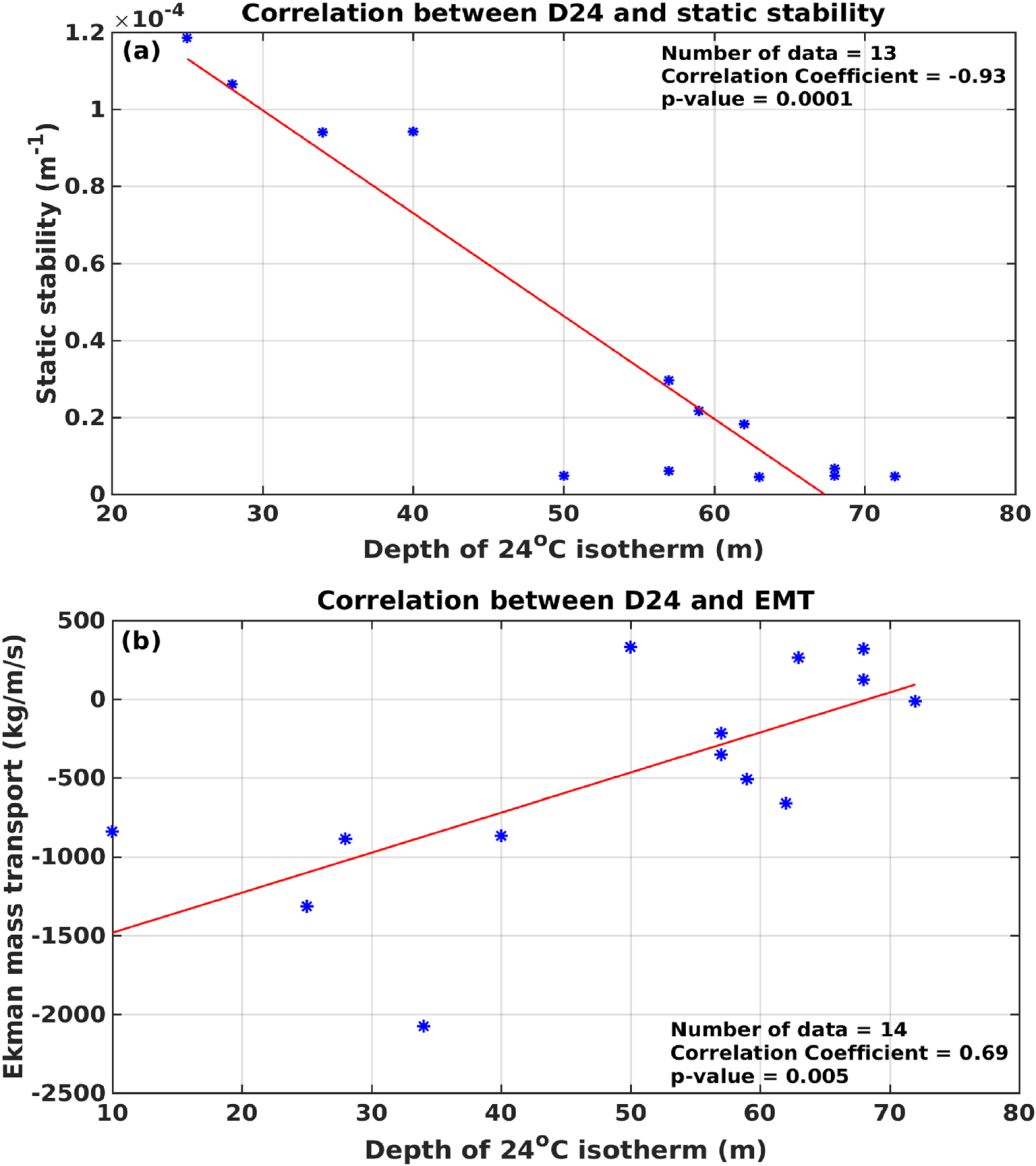
Correlation of depth of 24 °C isotherm (D_24_, m) with (**a**) static stability (m^−1^) in the upper 15 m of the water column, and (**b**) Ekman mass transport (EMT) (kg/m/s). All parameters were taken at 14 stations from 8° to 21° N.

Thus, based on the above we infer that the increased freshwater influx in the southern part of the EAS due to a combination of rainfall and subsequent river runoff along with the oceanic precipitation lead to an increase in the static stability of the upper water column along the coastal and shelf regions. This in turn increased the upper ocean stratification which led to the reduction of upwelling. In a wind-driven upwelling system, the EMT is often taken as an indicator of upwelling intensity^22,33^. To examine the role of stratification as well as wind during August 2017, the correlation of D_24_ with EMT and static stability were computed by taking values at stations from 8° to 21° N (Fig. 7). The static stability showed an inverse correlation with D_24,_ while the EMT showed a direct correlation. However, the correlation was stronger with D_24_ (r = − 0.93) than with EMT (r = 0.69) and both were significant at 99% confidence level (*p* values 0.0001 and 0.005 respectively). The results implied that both wind and stratification are important in regulating upwelling.

In the EAS, the upwelling intensity showed a considerable spatial variation as was the case during 2017. This spatial variation is driven by a combination of forcing such as wind and stratification, both of which showed considerable variability with latitude. The wind associated with the south-west monsoon has large spatial variability, and so also the rainfall. Recall that during August 2017 upwelling along the EAS was prominent at 11° N, which was a manifestation of the interplay between wind and stratification. The present study also showed that the rainfall, river discharge and stratification have considerable inter-annual variability. Hence, the spatial variation of the intensity of upwelling in the EAS would strongly depend on the relative dominance among the wind and the stratification.

### Role of stratification in regulating chlorophyll and small pelagic fishery

**Figure 8:**
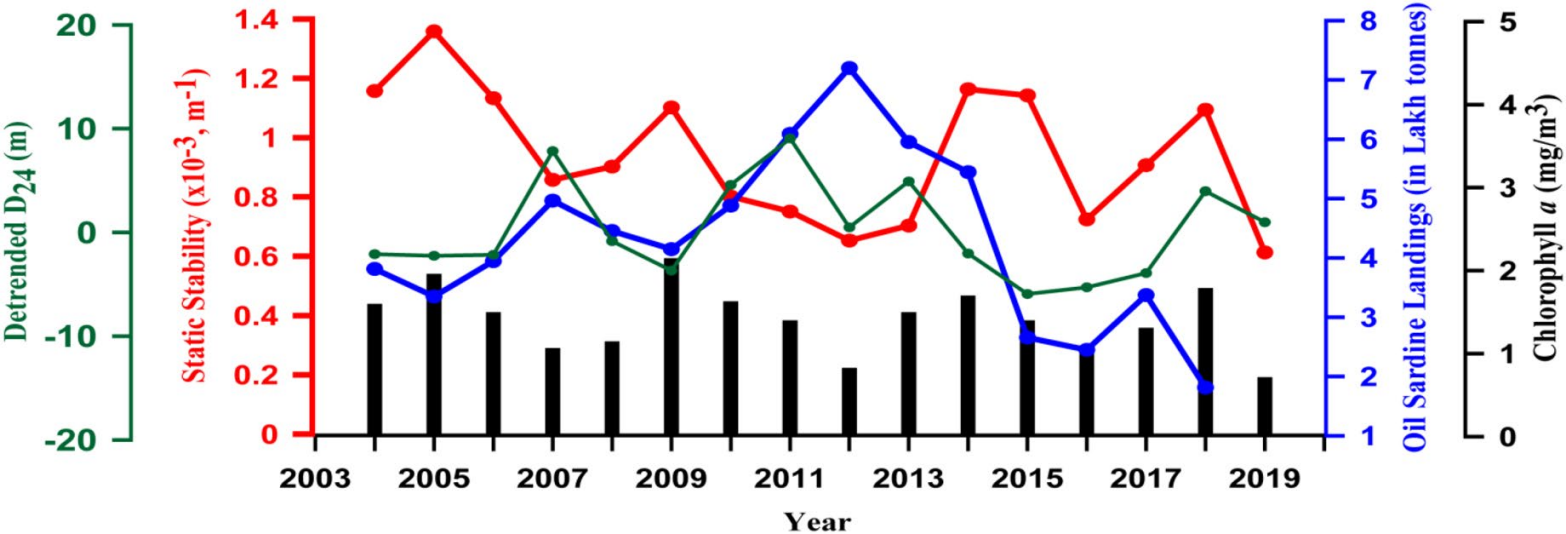
Time series of annual oil sardine landing (lakh tonnes, blue solid line), static stability (m^−1^, red solid line) in the upper 15 m of the water column, de-trended D_24_ (m, green solid line), and chlorophyll *a* (mg/m^3^, black solid line) from 2004 to 2019. The static stability, de-trended D_24,_ and the chlorophyll *a* were averaged over the boxes (8° to 12° N) and for the period from June to September. See Fig. 2 for the location of boxes.

Having examined the role of stratification in the process of upwelling in the EAS, we now focus on its impact on phytoplankton and fishery of this region. The oil sardines being dominant fish among the small pelagic fishery in the EAS^13,15^ we used the oil sardine landings data for our analysis. As the oil sardine landings from Kerala contribute nearly to 85% of the national oil sardine landings^40^, the national landings data would serve as a proxy for Kerala. Hence, the data on annual oil sardine landings (tonnes) available from 2004 to 2018 along with static stability (m^−1^), de-trended D_24_, and Chl-a (mg/m^3^) were examined (Fig. 8). The static stability, D_24,_ and Chl-a were averaged from 8° to 12° N and for the months June to September. The sardine landing data and static stability showed an inverse variation. For example, in the years 2005, 2009, 2015, and 2018 when the oil sardine landings were low, the static stability showed high values. Similarly, in the years 2007, 2012, and 2017 when the oil sardine landings were high, the static stability showed low values except in 2017. However, when the oil sardine landing crashed in 2015 we did not see a corresponding increase in static stability, but noticed a marginal decline. This is an anomaly which would need an explanation. An examination of ENSO (El Nino-Southern Oscillation) (https://origin.cpc.ncep.noaa.gov/products/analysis_monitoring/ensostuff/ONI_v5.php) and IOD (Indian Ocean Dipole) (http://www.bom.gov.au/climate/iod/) indices showed a co-occurrence of both in 2015. In fact, the year experienced one of the strongest El Nino (ENSO index 2.9) of the last two decades and a moderate positive IOD (index 0.7). During this combined event, the western Arabian Sea becomes warmer than normal and impacts upwelling. We speculate that the steep fall in oil sardine landing in 2015 was the manifestation of the co-occurrence of positive ENSO and IOD events.

A comparison of oil sardine landings with de-trended D_24_, however, showed a co-variation, except in 2012, 2013 and 2018, which is at variance with our expectation. The reason for the observed inverse variation of static stability and oil sardine landings lies in the fact that when the static stability of the water column is high, like that in 2017, upwelling is reduced due to strong stratification. This will restrict the nutrient input from subsurface to the upper ocean thereby reducing the chlorophyll biomass, which is a proxy for the phytoplankton biomass. Oil sardine being planktivorous fish^41^ a reduction in the phytoplankton biomass due to increased static stability (stratification) will reduce the biomass of oil sardine as well.

Hence, in an upwelling region like the EAS an inverse variation of Chl-a with static stability was expected. However, the results showed that the Chl-a co-varied with static stability (Fig. 8). For example, when static stability decreased during the periods from (1) 2005 to 2007, (2) 2009 to 2012, and (3) 2014 to 2016 the Chl-a also showed a decrease. Similarly, during the periods from (1) 2007 to 2009, (2) 2012 to 2014, and (3) 2016 to 2018 when the static stability showed an increase, the Chl-a also increased. This would need an explanation. Recall the presence of low salinity waters in the region from 8° to 13° N in the in situ observation (Fig. 3b), which is due to the freshwater influx in part by river run-off and in part by oceanic precipitation. The river run-off is capable of bringing along with it the land-derived nutrients into the coastal and near-shore waters and enhance the surface Chl-a. This is consistent with the earlier study^42^ in which it was found that riverine nutrients significantly contributed towards the increase of Chl-a, in addition to upwelling in the southern part of the EAS. Though nutrients brought by the freshwater increases the chlorophyll biomass, it is restricted to the thin surface layer, typically less than 20 m. The freshwater, however, will restrict upwelling through stratification and curtail the subsurface nutrient-driven chlorophyll enhancement. Upwelling-driven chlorophyll enhancement takes place within the euphotic zone, which is typically in the upper 50 m of the water column in the Arabian Sea^43^. The oil sardine being a planktivorous fish, it is the chlorophyll in the euphotic zone that sustains its biomass rather than the surface chlorophyll. This is the reason why there was no connection between the satellite derived Chl-a and the oil sardine landings. Instead, a close connection was noticed between static stability and oil sardine landing, a result which is not reported earlier.

To further substantiate the above result a multiple linear regression analysis was performed to assess the relationship between three predictors such as de-trended D_24_, static stability, and Chl-a with annual oil sardine landing as predictant. The predictors were ranked on the basis of the magnitude of their standardized regression coefficient presented in Table 2. The static stability was the most important predictor (ranked 1), followed by de-trended D_24_, both of which were significant at 95%. Chlorophyll was found to be the least important (ranked 3) among the predictors and was non-significant. This clearly indicated that the annual oil sardine catch is significantly and negatively correlated with static stability, while the annual oil sardine catch is significantly and positively correlated with de-trended D_24_. The multiple linear regression analysis confirms the inverse relationship between stratification and oil sardine landings, and reiterates the influence of environmental conditions on the habitat of planktivorous oil sardine^41,44^, which depends largely on the upwelling supported, nutrient-replete habitat^9^.

**Table 2:** Multiple linear regression analysis with annual oil sardine landings the predictand and parameters such as static stability, de-trended D_24_, and chlorophyll as predictors for the period 2004–2018.

Predictor	Rank	Regression coefficient	Standard error	*t*	*p* >|*t|*
Static Stability	1	− 0.524	0.236	− 2.216	0.045*
De-trended D_24_	2	0.496	0.224	2.001	0.050*
Chl-a	3	− 0.325	0.262	− 1.238	0.238

D_24_: Depth of 24 degree isotherm, Chl-a: chlorophyll-*a*. *denotes the values that are statistically significant at *p* < 0.05.

In order to establish the interdependencies among different parameters of upwelling and oil sardine, we calculated the partial correlation among nine variables viz. D_24_, static stability, EMT, river discharge, Kerala rainfall, oceanic precipitation, SST, Chl-a and oil sardine landings (Table 3). The D_24_ showed a high correlation with static stability (− 0.76), SST (0.73), and oil sardine landings (0.60). This reiterates our result that upwelling is inversely related to the static stability (Fig. 6), but co-varies with oil sardine landings (Fig. 8).

**Table 3:** Partial correlation among nine variables viz. D_24_, static stability, EMT, river discharge, Kerala rainfall, oceanic precipitation, SST, Chl-a and oil sardine landings.

Partial correlation	D_24_	Static stability	EMT	River discharge	Kerala Rainfall	Oceanic precipitation	SST	Chl-a	Oil Sardine landings (annual)
D_24_	1								
Static stability	− **0.76**	1							
EMT	0.40	0.01	1						
River discharge	0.19	− 0.01	0.20	1					
Kerala Rainfall	0.34	− 0.21	0.28	**0.85**	1				
Oceanic precipitation	**0.57**	**0.54**	0.36	**0.62**	**0.74**	1			
SST	**0.73**	**0.52**	**0.57**	− 0.11	0.14	0.42	1		
Chl-a	− 0.18	0.14	**0.57**	− 0.15	− 0.25	− 0.18	− 0.42	1	
Oil Sardine landings (annual)	**0.60**	− 0.43	0.43	0.19	0.17	0.42	0.34	− 0.22	1

All parameters were average during June to September over the boxes from 8 to 12° N. Valued > 0.5 is bold faced.

In summary, present study adds new information that the interannual variability of upwelling in the EAS is strongly linked to the interannual variation of fresh water influx via Kerala river discharge and oceanic precipitation. Further, a close connection between stratification and oil sardine landings was also deciphered.

**Figure 9:**
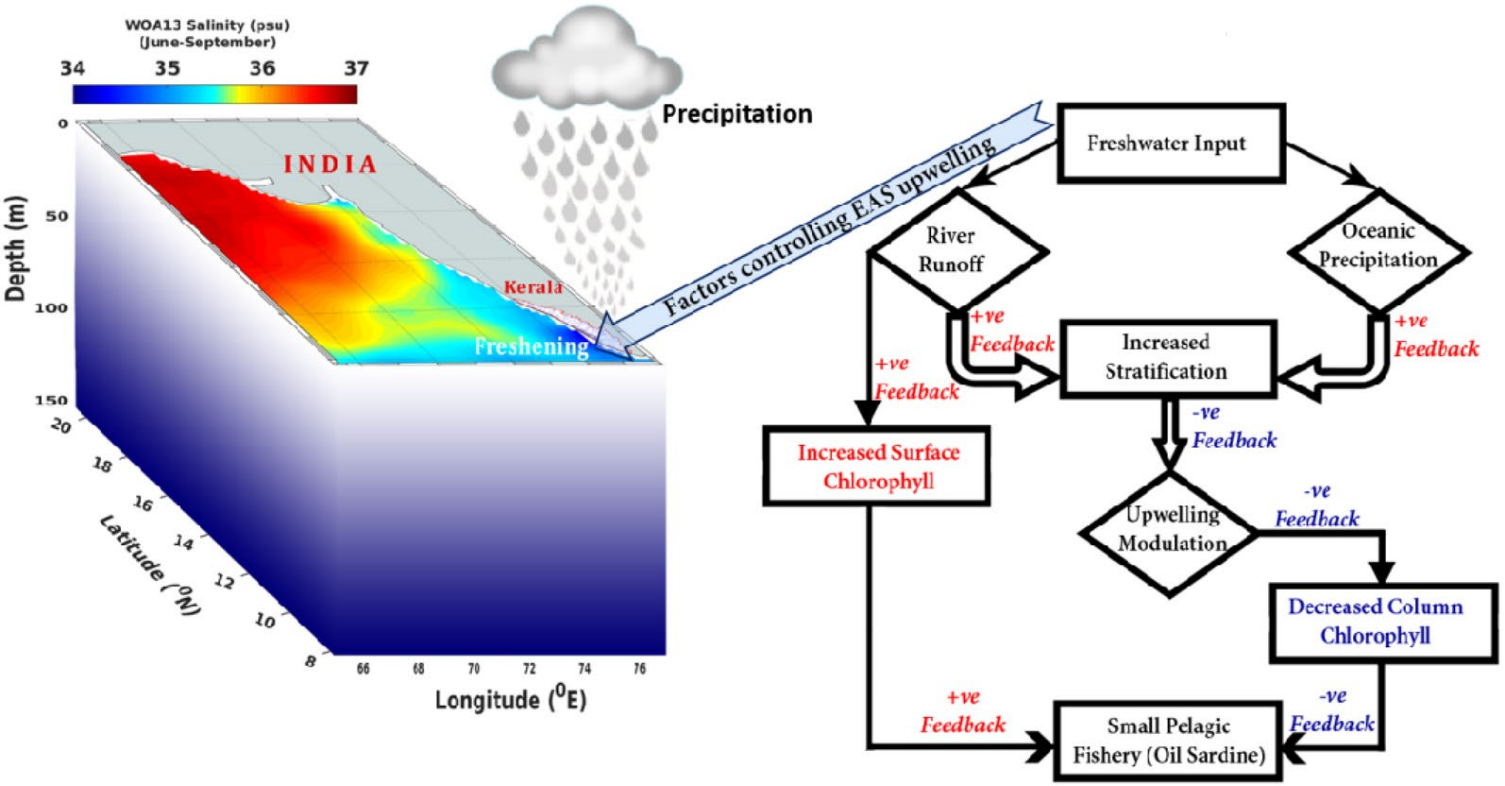
Schematics diagram depicting the mechanistic relationship among freshwater input, stratification, upwelling, chlorophyll, and oil sardine in the EAS. Figure was created using MATLAB and Adobe Photoshop software. See text for details.

The present study revealed that the surface chlorophyll need not always be an indicator of upwelling in the EAS and hence need not be tightly coupled to the small pelagic fishery. This is because the surface chlorophyll can be enhanced by the freshwater influx driven nutrient input, when upwelling is curtailed by stratification. The mechanistic relationship between the fresh water influx, stratification, upwelling, chlorophyll and oil sardine landings are summarised in the schematic diagram (Fig. 9).

The limitation of present study is the lack of data on nutrients, sub-surface chlorophyll, and fish biomass. In the absence of biomass data, though the oil sardine landings data serves as proxy for abundance, it is not the abundance per se. Fish landings data needs to be used with caution. Since oil sardines are fished using various combinations of craft and gear, the landings data are strongly dependent on the fish catch and the efforts put in.

## Data and methods

Upwelling in the EAS being a seasonal phenomenon active during June to September, it is important to capture the signature of upwelling when it is fully developed. As the peak upwelling occurs during July to August, multi-disciplinary in situ data was collected from EAS at stations from 8 to 21° N (Fig. 2) during August 2017 following the Joint Global Ocean Flux Studies (JGOFS) protocol^45^. The data was collected onboard ORV *Sindhu Sadhana* from 3^rd^ to 28^th^ August 2017. The Conductivity- Temperature-Depth (CTD) profiles from the surface to near-bottom up to 150 m were taken at 1-degree latitude interval using Sea-Bird Electronics CTD.

The temperature and salinity data obtained from the CTD were further used for the computation of static stability following^46^

1 $$E = \frac{ - 1}{\rho }\left[ {\frac{\partial \rho }{{\partial z}}} \right]$$

where E is the static stability (m^−1^), ρ is the density (kg m^−3^) of the water, and z is the depth (m).

The 3-day composite profile data of temperature and salinity were obtained from Estimating the Circulation and Climate of the Ocean- Jet Propulsion Laboratory (ECCO-JPL) ^47,48^ having a spatial resolution of 0.5 × 0.5 degree for the years from 2001 to 2019 (http://apdrc.soest.hawaii.edu/las/v6/constrain?var=5023). Monthly values of temperature and salinity were calculated from 3-day composite profile. This was further used for the computation of depth of 24 °C isotherm (D_24_) and static stability at station locations from 8° to 12° N for 2001 to 2019.

The climatological monthly mean data on temperature^49^ and salinity^50^ for August having a spatial resolution of 0.25 × 0.25 degrees was obtained from World Ocean Atlas 2013 (WOA13) (http://apdrc.soest.hawaii.edu/las/v6/dataset?catitem=21428).

The monthly mean SST data for the 2017 was obtained from National Oceanic and Atmospheric Administration (NOAA) Extended Reconstructed SST (ERSST) (http://apdrc.soest.hawaii.edu/las/v6/constrain?var=1262)^51^.

The monthly mean Chl-a pigment concentration having a spatial resolution of 4 km (zonal) × 4 km (meridional) was obtained from Copernicus-GlobColour using the website https://resources.marine.copernicus.eu/?option=com_csw&view=order&record_id=918b8402-78b1-4236-b11e-8202085f0159 which is a merged product of sensors such as SeaWiFS, MODIS, MERIS, VIIRS-SNPP&JPSS1, OLCI-S#A&S#B^52^. This was used for the computation of seasonal mean during June to September for each year and the climatological seasonal mean for the period 2001 to 2019.

The monthly mean geostrophic current having a spatial resolution of 0.5 × 0.5 degree for August 2017 was obtained from Copernicus-Marine Environment Monitoring Service (CMEMS) (https://resources.marine.copernicus.eu/?option=com_csw&task=results?option=com_csw&view=details&product_id=SEALEVEL_GLO_PHY_L4_REP_OBSERVATIONS_008_047).

The 6-hourly data of surface winds, zonal (u) and meridional components (v), were taken from the Morden Era Retrospective analysis for Research and Application (MERRA)^53^ having a 0.66 × 0.5 degree spatial resolution (http://apdrc.soest.hawaii.edu/las/v6/dataset?catitem=17656). Using u and v the zonal and meridional wind stress were calculated using following equations

2 $$\tau_{x} = \rho C_{D} U_{10} u$$

3 $$\tau_{y} = \rho C_{D} U_{10} v$$

where ρ is the density of air taken as 1.22 kg m^−3^, C_D_ is the dimensionless drag coefficient and U_10_ is the speed of wind at 10 m above the sea surface. The value of drag coefficient that varies with wind speed as per Large and Pond^54^ were used. Further, the Ekman vertical velocity^55^ and Ekman mass transport^56^ (EMT) were computed following equations

4 $$Ekman\,Vertical\,velocity = \frac{ - 1}{{\rho f}}\left( {\frac{{\partial \tau_{y} }}{\partial x} - \frac{{\partial \tau_{x} }}{\partial y}} \right)$$

5 $$M_{e} = \frac{{\tau_{y} }}{f}$$

where $\tau_{x}$ and $\tau_{y}$ are the zonal and meridional wind stress components, *ρ* is the density of seawater taken as 1026 kg m^−3^, and *f* is the Coriolis parameter and $M_{e}$ is the EMT in kg/m/s.

Monthly data on precipitation having a spatial resolution of 0.5 × 0.5 degrees for the years 2001 to 2019 were extracted from tropical rainfall measuring mission (TRMM)^57^ (http://apdrc.soest.hawaii.edu/las/v6/constrain?var=13346).

The monthly rainfall data for the state of Kerala for the period 2001 to 2019 was obtained from Indian Institute of Tropical Meteorology (http://www.tropmet.res.in/static_page.php?page_id=53) and Kerala river discharge (2001 to 2018) from Department of water resources, Govt. of India Water Resources Information System (INDIA-WRIS) (http://indiawris.gov.in).

The annual national oil sardine landing (tonnes) data available during 2004 to 2018 were obtained from Central Marine Fisheries Research Institute (CMFRI) (www.cmfri.org.in/fish-catch/estimates).

## Data Availability

All the data used in the study were downloaded from the open source and the web site details are given under Data and Method. The in situ data on temperature and salinity collected during August 2017 is available with the data repository at CSIR-NIO (https://www.nio.org).
